# Crystal structure of *trans*-(1,8-dibutyl-1,3,6,8,10,13-hexa­aza­cyclo­tetra­decane-κ^4^ *N* ^3^,*N* ^6^,*N* ^10^,*N* ^13^)bis­(isonicotinato-κ*O*)nickel(II) determined from synchrotron data

**DOI:** 10.1107/S2056989016001031

**Published:** 2016-01-23

**Authors:** Jong Won Shin, Dae-Woong Kim, Dohyun Moon

**Affiliations:** a Beamline Department, Pohang Accelerator Laboratory 80, Jigokro-127-beongil, Nam-Gu Pohang, Gyeongbuk 37673, Republic of Korea

**Keywords:** crystal structure, aza­macrocyclic ligand, isonicotinic acid, π–π inter­actions, synchrotron data

## Abstract

The title compound, [Ni(C_6_H_4_NO_2_)_2_(C_16_H_38_N_6_)], was prepared through self-assembly of a nickel(II) aza­macrocyclic complex with isonicotinic acid. The Ni^II^ atom is located on an inversion center and exhibits a distorted octa­hedral N_4_O_2_ coordination environment, with the four secondary N atoms of the aza­macrocyclic ligand in the equatorial plane [average Ni—N_eq_ = 2.064 (11) Å] and two O atoms of monodentate isonicotinate anions in axial positions [Ni—O_ax_ = 2.137 (1) Å]. Intra­molecular N—H⋯O hydrogen bonds between one of the secondary amine N atoms of the aza­macrocyclic ligand and the non-coordinating carboxyl­ate O atom of the anion stabilize the mol­ecular structure. Inter­molecular N—H⋯N hydrogen bonds, as well as π–π inter­actions between neighbouring pyridine rings, give rise to the formations of supra­molecular ribbons extending parallel to [001].

## Abstract 2

The Ni^II^ atom in the title compound shows a slightly distorted octa­hedral coordination environment to four N atoms of the aza­macrocylic ligand in the equatorial plane and two isonicotinate O atoms in axial positions. Inter­molecular N—H⋯N hydrogen bonds and π–π inter­actions consolidate the crystal packing.

## Chemical context   

The mol­ecular design and synthesis of coordination polymers with macrocyclic ligands have attracted considerable attention because of their potential applications in chemistry, environmental chemistry, and materials science (Churchard *et al.*, 2010 ▸; Lehn, 2015 ▸). To obtain specific mol­ecular compounds through assembly of supra­molecular building blocks with properties such as guest recognition or catalytic effects, macrocyclic complexes involving vacant sites in an axial position are good candidates. Moreover, these complexes can also be easily derivatized by carb­oxy­lic acid moieties, such as 1,3,5-BTC (1,3,5-benzene­tri­carb­oxy­lic acid), 2,7-NDC (2,7-naphthalenedi­carb­oxy­lic acid) or 1,3,5-CTC (1,3,5-cyclo­hexa­netri­carb­oxy­lic acid), forming inter­esting coordination compounds with supra­molecular structures ranging from chains to networks (Min & Suh, 2001 ▸; Shin *et al.*, 2016*b* ▸). For example, [Ni(*L^R^*,^*R*^)]_3_[BTC^3–^]_2_·12H_2_O·CH_3_CN (*L^R^*,^*R*^ = 1,8-bis­[(*R*)-α-methyl­benz­yl]-1,3,6,8,10,13-hexa­aza­cyclo­tetra­deca­ne) displays a two-dimensional supra­molecular network structure and exhibits a selective chiral recognition for racemic material (Ryoo *et al.*, 2010 ▸). Isonicotinic acid as another building unit can easily bind or inter­act with transition metal ions through its possible bridging or coordination modes associated with the carb­oxy­lic group and pyridine moieties, respectively, thus allowing the assembly of compounds with supra­molecular structures or the formation of heterometallic complexes (Xie *et al.*, 2014 ▸).

Here, we report on the synthesis and crystal structure of an Ni^II^ aza­macrocyclic complex including isonicotinate anions, [Ni(C_6_H_4_NO_2_)_2_(C_16_H_38_N_6_)], (**I**).
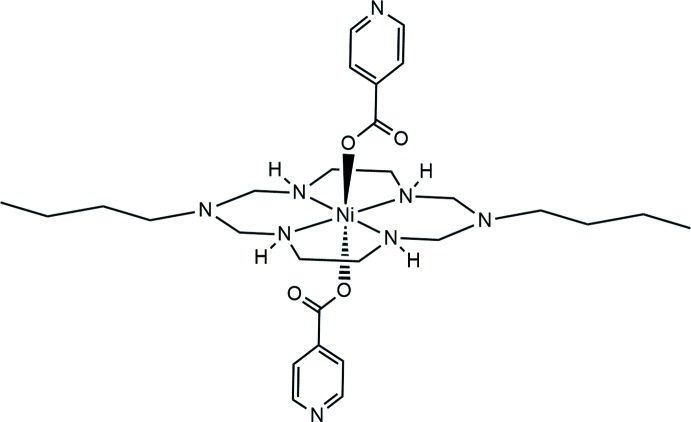


## Structural commentary   

**Figure 1:**
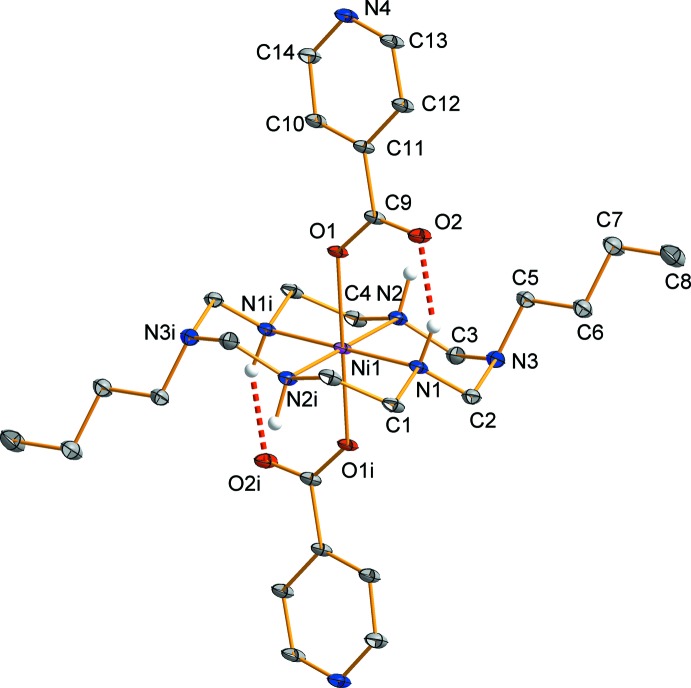
View of the mol­ecular structure of the title compound, showing the atom-labelling scheme, with displacement ellipsoids drawn at the 30% probability level. H atoms bonded to C atoms have been omitted for clarity. Intra­molecular N—H⋯O hydrogen bonds are shown as red dashed lines. [Symmetry code: (i) −*x* + 1, −*y* + 1, −*z* + 1.]

Compound (**I**) is isotypic with its copper(II) analogue (Shin *et al.*, 2015 ▸). The nickel(II) atom is located on an inversion center. The coordination environment around the nickel(II) atom is distorted octa­hedral with the four secondary amine N atoms of the aza­macrocyclic ligand in the equatorial plane and two O atoms of two monodentate isonicotinate anions in axial positions (Fig. 1 ▸). The average Ni—N_eq_ bond lengths is 2.064 (11) Å and the Ni—O_ax_ bond length is 2.137 (1) Å. The longer axial bonds can be attributed to a ring contraction of the aza­macrocyclic ligand (Melson, 1979 ▸). The six-membered NiC_2_N_3_ ring (Ni1–N1–C2–N3–C3–N2) adopts the expected chair conformation, whereas the five-membered NiC_2_N_2_ ring (Ni1–N1–C1–C4–N2) has a *gauche* conformation (Min & Suh, 2001 ▸). Since the carboxyl­ate group is fully delocalized, the two C—O bonds and the bond angle (O1—C9—O2) are 1.267 (2), 1.248 (2) Å and 126.9 (2)°, respectively. The bond angles around the nickel(II) atom are in the normal range for an octa­hedral complex. Intra­molecular N—H⋯O hydrogen bonds between one of the secondary amine groups of the aza­macrocyclic ligand and the non-coordinating carboxyl­ate O atom of the isonicotinate anion form six-membered rings and stabilize the mol­ecular structure (Fig. 1 ▸, Table 1 ▸).

## Supra­molecular features   

**Figure 2:**
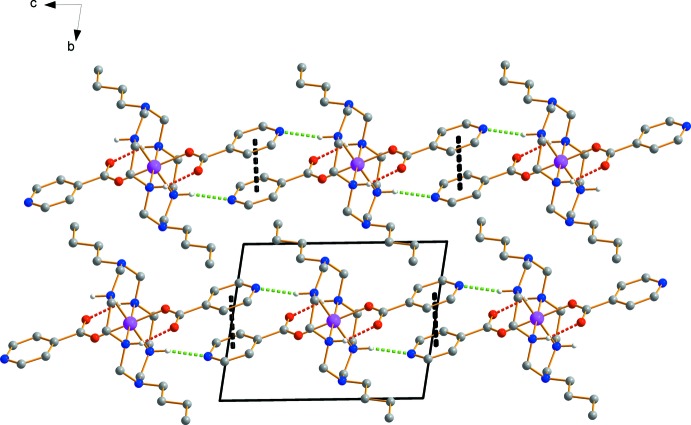
View of the crystal packing of the title compound, showing hydrogen bonds and π–π inter­actions (red: intra­molecular N—H⋯O hydrogen bonds, green: inter­molecular N—H⋯N hydrogen bonds, black: π–π inter­actions).

The N4 atom of the isonicotinate anion forms an inter­molecular hydrogen bond with an adjacent secondary amine group of the aza­macrocyclic ligand (Fig. 2 ▸, Table 1 ▸) (Steed & Atwood, 2009 ▸). In addition, parallel pyridine rings (Hunter & Sanders, 1990 ▸) of the isonicotinate anions participate in π–π inter­actions with a centroid-to-centroid distance of 3.741 (1) Å and an inter­planar separation of 3.547 (1) Å. The inter­play between hydrogen bonds and π–π inter­actions give rise to the formation of supra­molecular ribbons extending parallel to [001].

## Database survey   

A search of the Cambridge Structural Database (Version 5.36, May 2014 with 3 updates; Groom & Allen, 2014 ▸) reveals two complexes with the same nickel(II) aza­macrocyclic building block (Kim *et al.*, 2015**a* ▸,b* ▸) for which synthesis, FT–IR spectroscopic data and the crystal structure have been reported.

## Synthesis and crystallization   

The starting complex, [Ni(C_16_H_38_N_6_)(ClO_4_)_2_], was prepared in a slightly modified procedure with respect to the reported method (Kim *et al.*, 2015*b* ▸). To an aceto­nitrile solution (14 mL) of [Ni(C_16_H_38_N_6_)(ClO_4_)_2_] (0.298 g, 0.52 mmol) was slowly added an aceto­nitrile solution (8 mL) containing isonicotinic acid (0.128 g, 1.04 mmol) and excess tri­ethyl­amine (0.12 g, 1.20 mmol) at room temperature. The purple precipitate was filtered off, washed with aceto­nitrile and diethyl ether, and dried in air. Single crystals of compound (**l**) were obtained by layering of the aceto­nitrile solution of isonicotinic acid on the aceto­nitrile solution of [Ni(C_16_H_38_N_6_)(ClO_4_)_2_] for several days. Yield: 0.167 g (52%). FT–IR (ATR, cm^−1^): 3145, 3075, 2951, 2920, 1571, 1457, 1351, 1272, 1014, 915.

*Safety note:* Although we have experienced no problems with the compounds reported in this study, perchlorate salts of metal complexes are often explosive and should be handled with great caution.

## Refinement   

Crystal data, data collection and structure refinement details are summarized in Table 2 ▸. All H atoms were placed in geometrically idealized positions and constrained to ride on their parent atoms, with C—H distances of 0.95 (ring H atoms) or 0.98–0.99 Å (open-chain H atoms), and an N—H distance of 1.0 Å, with *U*_iso_(H) values of 1.2 or 1.5*U*_eq_ of the parent atoms.

## Supplementary Material

Crystal structure: contains datablock(s) I. DOI: 10.1107/S2056989016001031/wm5263sup1.cif

Structure factors: contains datablock(s) I. DOI: 10.1107/S2056989016001031/wm5263Isup2.hkl

CCDC reference: 1447865

Additional supporting information:  crystallographic information; 3D view; checkCIF report

## Figures and Tables

**Table 1:** Hydrogen-bond geometry (Å, °)

*D*—H⋯*A*	*D*—H	H⋯*A*	*D*⋯*A*	*D*—H⋯*A*
N1—H1⋯O2	1.00	1.98	2.892 (2)	150
N2—H2⋯N4^i^	1.00	2.23	3.143 (2)	151

Symmetry code: (i) 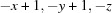.

**Table 2:** Experimental details

Crystal data	
Chemical formula	[Ni(C_6_H_4_NO_2_)_2_(C_16_H_38_N_6_)]
*M*_r_	617.44
Crystal system, space group	Triclinic, *P* 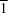
Temperature (K)	100
*a*, *b*, *c* (Å)	8.0630 (16), 8.5110 (17), 10.927 (2)
α, β, γ (°)	80.52 (3), 88.26 (3), 86.44 (3)
*V* (Å^3^)	738.0 (3)
*Z*	1
Radiation type	Synchrotron, λ = 0.62998 Å
μ (mm^−1^)	0.51
Crystal size (mm)	0.01 × 0.004 × 0.004

Data collection	
Diffractometer	ADSC Q210 CCD area detector
Absorption correction	Empirical (*HKL-3000SM *SCALEPACK**; Otwinowski & Minor, 1997 ▸)
*T*_min_, *T*_max_	0.995, 0.998
No. of measured, independent and observed [*I* > 2σ(*I*)] reflections	7634, 3879, 3326
*R*_int_	0.023
(sin θ/λ)_max_ (Å^−1^)	0.696

Refinement	
*R*[*F* ^2^ > 2σ(*F* ^2^)], *wR*(*F* ^2^), *S*	0.040, 0.110, 1.04
No. of reflections	3879
No. of parameters	188
H-atom treatment	H-atom parameters constrained
Δρ_max_, Δρ_min_ (e Å^−3^)	1.12, −0.95

Computer programs: *PAL BL2D-SMDC* (Shin *et al.*, 2016 ▸ *a*), *HKL-3000SM* (Otwinowski & Minor, 1997 ▸), *SHELXT* (Sheldrick, 2015*a* ▸), *SHELXL2014* (Sheldrick, 2015*b* ▸), *DIAMOND* (Putz & Brandenburg, 2014 ▸) and *publCIF* (Westrip, 2010 ▸).

## supplementary crystallographic information

### Crystal data

**Table:**

[Ni(C_6_H_4_NO_2_)_2_(C_16_H_38_N_6_)]	*Z* = 1
*M**_r_* = 617.44	*F*(000) = 330
Triclinic, *P*1	*D*_x_ = 1.389 Mg m^−^^3^
*a* = 8.0630 (16) Å	Synchrotron radiation, λ = 0.62998 Å
*b* = 8.5110 (17) Å	Cell parameters from 20128 reflections
*c* = 10.927 (2) Å	θ = 0.4–33.6°
α = 80.52 (3)°	µ = 0.51 mm^−^^1^
β = 88.26 (3)°	*T* = 100 K
γ = 86.44 (3)°	Needle, pale pink
*V* = 738.0 (3) Å^3^	0.01 × 0.004 × 0.004 mm

### Data collection

**Table:**

ADSC Q210 CCD area-detector diffractometer	3326 reflections with *I* > 2σ(*I*)
Radiation source: PLSII 2D bending magnet	*R*_int_ = 0.023
ω scan	θ_max_ = 26.0°, θ_min_ = 2.5°
Absorption correction: empirical (using intensity measurements) (*HKL-3000SM *SCALEPACK**; Otwinowski & Minor, 1997)	*h* = −11→11
*T*_min_ = 0.995, *T*_max_ = 0.998	*k* = −11→11
7634 measured reflections	*l* = −15→15
3879 independent reflections	

### Refinement

**Table:**

Refinement on *F*^2^	0 restraints
Least-squares matrix: full	Hydrogen site location: inferred from neighbouring sites
*R*[*F*^2^ > 2σ(*F*^2^)] = 0.040	H-atom parameters constrained
*wR*(*F*^2^) = 0.110	*w* = 1/[σ^2^(*F*_o_^2^) + (0.0649*P*)^2^ + 0.1918*P*] where *P* = (*F*_o_^2^ + 2*F*_c_^2^)/3
*S* = 1.04	(Δ/σ)_max_ < 0.001
3879 reflections	Δρ_max_ = 1.12 e Å^−^^3^
188 parameters	Δρ_min_ = −0.95 e Å^−^^3^

### Special details

**Table:**

Geometry. All e.s.d.'s (except the e.s.d. in the dihedral angle between two l.s. planes) are estimated using the full covariance matrix. The cell e.s.d.'s are taken into account individually in the estimation of e.s.d.'s in distances, angles and torsion angles; correlations between e.s.d.'s in cell parameters are only used when they are defined by crystal symmetry. An approximate (isotropic) treatment of cell e.s.d.'s is used for estimating e.s.d.'s involving l.s. planes.

### Fractional atomic coordinates and isotropic or equivalent isotropic displacement parameters (Å^2^)

**Table:**

	*x*	*y*	*z*	*U*_iso_*/*U*_eq_	
Ni1	0.5000	0.5000	0.5000	0.02066 (10)	
O1	0.43513 (15)	0.41029 (17)	0.33736 (11)	0.0257 (3)	
O2	0.18613 (16)	0.52908 (19)	0.28022 (12)	0.0322 (3)	
N1	0.27809 (18)	0.6311 (2)	0.50735 (13)	0.0244 (3)	
H1	0.2171	0.6276	0.4296	0.029*	
N2	0.61452 (18)	0.67953 (19)	0.38249 (13)	0.0241 (3)	
H2	0.5798	0.6766	0.2959	0.029*	
N3	0.3946 (2)	0.8835 (2)	0.41256 (14)	0.0304 (3)	
N4	0.3686 (2)	0.2837 (2)	−0.09149 (14)	0.0335 (4)	
C1	0.1835 (2)	0.5457 (3)	0.61221 (15)	0.0276 (4)	
H1A	0.0642	0.5807	0.6057	0.033*	
H1B	0.2242	0.5692	0.6915	0.033*	
C2	0.3004 (2)	0.8006 (2)	0.51521 (16)	0.0296 (4)	
H2A	0.1895	0.8566	0.5192	0.036*	
H2B	0.3574	0.8058	0.5933	0.036*	
C3	0.5703 (2)	0.8414 (2)	0.41070 (18)	0.0308 (4)	
H3A	0.6153	0.8493	0.4926	0.037*	
H3B	0.6249	0.9202	0.3480	0.037*	
C4	0.7938 (2)	0.6330 (3)	0.39084 (16)	0.0280 (4)	
H4A	0.8376	0.6572	0.4689	0.034*	
H4B	0.8552	0.6932	0.3203	0.034*	
C5	0.3160 (2)	0.9034 (2)	0.29077 (17)	0.0310 (4)	
H5A	0.2983	0.7968	0.2700	0.037*	
H5B	0.3922	0.9575	0.2269	0.037*	
C6	0.1502 (3)	0.9999 (3)	0.28701 (18)	0.0341 (4)	
H6A	0.0727	0.9447	0.3493	0.041*	
H6B	0.1671	1.1058	0.3093	0.041*	
C7	0.0730 (3)	1.0223 (3)	0.15954 (19)	0.0390 (5)	
H7A	0.1527	1.0726	0.0966	0.047*	
H7B	0.0507	0.9166	0.1390	0.047*	
C8	−0.0882 (3)	1.1257 (4)	0.1543 (2)	0.0502 (6)	
H8A	−0.1692	1.0739	0.2140	0.075*	
H8B	−0.1328	1.1395	0.0705	0.075*	
H8C	−0.0667	1.2302	0.1750	0.075*	
C9	0.3163 (2)	0.4477 (2)	0.26295 (15)	0.0235 (3)	
C10	0.4760 (2)	0.2978 (3)	0.10910 (16)	0.0294 (4)	
H10	0.5631	0.2691	0.1666	0.035*	
C11	0.3367 (2)	0.3884 (2)	0.13963 (14)	0.0234 (3)	
C12	0.2134 (2)	0.4252 (3)	0.05120 (16)	0.0300 (4)	
H12	0.1158	0.4874	0.0676	0.036*	
C13	0.2347 (2)	0.3703 (3)	−0.06044 (17)	0.0339 (4)	
H13	0.1486	0.3957	−0.1191	0.041*	
C14	0.4868 (2)	0.2496 (3)	−0.00601 (17)	0.0340 (4)	
H14	0.5838	0.1887	−0.0256	0.041*	

### Atomic displacement parameters (Å^2^)

**Table:**

	*U*^11^	*U*^22^	*U*^33^	*U*^12^	*U*^13^	*U*^23^
Ni1	0.01767 (15)	0.03464 (19)	0.00987 (14)	0.00142 (11)	−0.00201 (9)	−0.00504 (11)
O1	0.0246 (6)	0.0412 (7)	0.0122 (5)	0.0012 (5)	−0.0054 (4)	−0.0073 (5)
O2	0.0235 (6)	0.0543 (9)	0.0207 (6)	0.0047 (6)	−0.0043 (5)	−0.0139 (6)
N1	0.0225 (7)	0.0384 (8)	0.0122 (6)	0.0027 (6)	−0.0022 (5)	−0.0048 (6)
N2	0.0227 (7)	0.0355 (8)	0.0142 (6)	0.0007 (6)	−0.0016 (5)	−0.0049 (6)
N3	0.0338 (8)	0.0354 (9)	0.0209 (7)	0.0055 (7)	−0.0018 (6)	−0.0041 (7)
N4	0.0344 (8)	0.0525 (11)	0.0148 (6)	0.0002 (7)	−0.0027 (6)	−0.0091 (7)
C1	0.0186 (7)	0.0485 (11)	0.0149 (7)	0.0026 (7)	0.0012 (5)	−0.0052 (7)
C2	0.0331 (9)	0.0384 (10)	0.0169 (8)	0.0078 (8)	−0.0008 (6)	−0.0068 (7)
C3	0.0339 (9)	0.0350 (10)	0.0240 (8)	−0.0011 (7)	−0.0018 (7)	−0.0063 (8)
C4	0.0205 (8)	0.0460 (11)	0.0173 (7)	−0.0038 (7)	0.0002 (6)	−0.0043 (7)
C5	0.0376 (10)	0.0345 (10)	0.0188 (8)	0.0059 (8)	−0.0014 (7)	−0.0011 (7)
C6	0.0356 (10)	0.0414 (11)	0.0227 (9)	0.0064 (8)	−0.0008 (7)	−0.0009 (8)
C7	0.0394 (11)	0.0506 (13)	0.0243 (9)	0.0048 (9)	−0.0033 (7)	−0.0006 (9)
C8	0.0400 (12)	0.0733 (18)	0.0320 (11)	0.0119 (11)	−0.0033 (9)	0.0018 (11)
C9	0.0213 (7)	0.0366 (9)	0.0128 (7)	−0.0044 (6)	−0.0018 (5)	−0.0040 (6)
C10	0.0258 (8)	0.0468 (11)	0.0160 (7)	0.0028 (7)	−0.0044 (6)	−0.0071 (7)
C11	0.0222 (7)	0.0365 (9)	0.0119 (7)	−0.0038 (7)	−0.0016 (5)	−0.0047 (7)
C12	0.0257 (8)	0.0484 (11)	0.0158 (7)	0.0020 (8)	−0.0044 (6)	−0.0062 (8)
C13	0.0307 (9)	0.0562 (13)	0.0157 (8)	0.0009 (8)	−0.0073 (6)	−0.0085 (8)
C14	0.0306 (9)	0.0534 (12)	0.0186 (8)	0.0056 (8)	−0.0017 (7)	−0.0106 (8)

### Geometric parameters (Å, º)

**Table:**

Ni1—N1^i^	2.0559 (16)	C3—H3B	0.9900
Ni1—N1	2.0559 (16)	C4—C1^i^	1.526 (3)
Ni1—N2	2.0720 (17)	C4—H4A	0.9900
Ni1—N2^i^	2.0720 (17)	C4—H4B	0.9900
Ni1—O1^i^	2.1371 (13)	C5—C6	1.523 (3)
Ni1—O1	2.1372 (13)	C5—H5A	0.9900
O1—C9	1.2669 (19)	C5—H5B	0.9900
O2—C9	1.248 (2)	C6—C7	1.521 (3)
N1—C1	1.471 (2)	C6—H6A	0.9900
N1—C2	1.481 (3)	C6—H6B	0.9900
N1—H1	1.0000	C7—C8	1.521 (3)
N2—C4	1.477 (2)	C7—H7A	0.9900
N2—C3	1.481 (3)	C7—H7B	0.9900
N2—H2	1.0000	C8—H8A	0.9800
N3—C3	1.440 (3)	C8—H8B	0.9800
N3—C2	1.444 (2)	C8—H8C	0.9800
N3—C5	1.471 (2)	C9—C11	1.516 (2)
N4—C13	1.336 (3)	C10—C14	1.384 (3)
N4—C14	1.340 (2)	C10—C11	1.386 (3)
C1—C4^i^	1.526 (3)	C10—H10	0.9500
C1—H1A	0.9900	C11—C12	1.393 (2)
C1—H1B	0.9900	C12—C13	1.379 (3)
C2—H2A	0.9900	C12—H12	0.9500
C2—H2B	0.9900	C13—H13	0.9500
C3—H3A	0.9900	C14—H14	0.9500
			
N1^i^—Ni1—N1	180.0	H3A—C3—H3B	107.6
N1^i^—Ni1—N2	85.97 (6)	N2—C4—C1^i^	108.14 (15)
N1—Ni1—N2	94.03 (6)	N2—C4—H4A	110.1
N1^i^—Ni1—N2^i^	94.03 (6)	C1^i^—C4—H4A	110.1
N1—Ni1—N2^i^	85.97 (6)	N2—C4—H4B	110.1
N2—Ni1—N2^i^	180.0	C1^i^—C4—H4B	110.1
N1^i^—Ni1—O1^i^	93.29 (6)	H4A—C4—H4B	108.4
N1—Ni1—O1^i^	86.71 (6)	N3—C5—C6	112.79 (16)
N2—Ni1—O1^i^	92.90 (6)	N3—C5—H5A	109.0
N2^i^—Ni1—O1^i^	87.10 (6)	C6—C5—H5A	109.0
N1^i^—Ni1—O1	86.71 (6)	N3—C5—H5B	109.0
N1—Ni1—O1	93.29 (6)	C6—C5—H5B	109.0
N2—Ni1—O1	87.10 (6)	H5A—C5—H5B	107.8
N2^i^—Ni1—O1	92.90 (6)	C7—C6—C5	111.93 (17)
O1^i^—Ni1—O1	180.0	C7—C6—H6A	109.2
C9—O1—Ni1	131.99 (12)	C5—C6—H6A	109.2
C1—N1—C2	114.34 (14)	C7—C6—H6B	109.2
C1—N1—Ni1	105.52 (11)	C5—C6—H6B	109.2
C2—N1—Ni1	112.75 (11)	H6A—C6—H6B	107.9
C1—N1—H1	108.0	C8—C7—C6	111.81 (19)
C2—N1—H1	108.0	C8—C7—H7A	109.3
Ni1—N1—H1	108.0	C6—C7—H7A	109.3
C4—N2—C3	113.96 (15)	C8—C7—H7B	109.3
C4—N2—Ni1	104.76 (11)	C6—C7—H7B	109.3
C3—N2—Ni1	113.72 (11)	H7A—C7—H7B	107.9
C4—N2—H2	108.0	C7—C8—H8A	109.5
C3—N2—H2	108.0	C7—C8—H8B	109.5
Ni1—N2—H2	108.0	H8A—C8—H8B	109.5
C3—N3—C2	115.84 (15)	C7—C8—H8C	109.5
C3—N3—C5	114.58 (15)	H8A—C8—H8C	109.5
C2—N3—C5	115.55 (16)	H8B—C8—H8C	109.5
C13—N4—C14	116.05 (17)	O2—C9—O1	126.88 (16)
N1—C1—C4^i^	108.60 (14)	O2—C9—C11	117.12 (15)
N1—C1—H1A	110.0	O1—C9—C11	115.99 (16)
C4^i^—C1—H1A	110.0	C14—C10—C11	119.30 (17)
N1—C1—H1B	110.0	C14—C10—H10	120.4
C4^i^—C1—H1B	110.0	C11—C10—H10	120.4
H1A—C1—H1B	108.4	C10—C11—C12	117.25 (16)
N3—C2—N1	114.07 (15)	C10—C11—C9	122.61 (15)
N3—C2—H2A	108.7	C12—C11—C9	120.14 (16)
N1—C2—H2A	108.7	C13—C12—C11	119.15 (18)
N3—C2—H2B	108.7	C13—C12—H12	120.4
N1—C2—H2B	108.7	C11—C12—H12	120.4
H2A—C2—H2B	107.6	N4—C13—C12	124.30 (17)
N3—C3—N2	114.51 (16)	N4—C13—H13	117.8
N3—C3—H3A	108.6	C12—C13—H13	117.8
N2—C3—H3A	108.6	N4—C14—C10	123.94 (18)
N3—C3—H3B	108.6	N4—C14—H14	118.0
N2—C3—H3B	108.6	C10—C14—H14	118.0
			
C2—N1—C1—C4^i^	−166.23 (14)	C5—C6—C7—C8	−177.2 (2)
Ni1—N1—C1—C4^i^	−41.74 (15)	Ni1—O1—C9—O2	−15.1 (3)
C3—N3—C2—N1	−72.6 (2)	Ni1—O1—C9—C11	164.15 (12)
C5—N3—C2—N1	65.5 (2)	C14—C10—C11—C12	0.4 (3)
C1—N1—C2—N3	179.49 (14)	C14—C10—C11—C9	−179.18 (18)
Ni1—N1—C2—N3	58.94 (17)	O2—C9—C11—C10	179.64 (18)
C2—N3—C3—N2	70.3 (2)	O1—C9—C11—C10	0.3 (3)
C5—N3—C3—N2	−68.2 (2)	O2—C9—C11—C12	0.1 (3)
C4—N2—C3—N3	−175.30 (14)	O1—C9—C11—C12	−179.24 (17)
Ni1—N2—C3—N3	−55.32 (18)	C10—C11—C12—C13	0.3 (3)
C3—N2—C4—C1^i^	167.84 (13)	C9—C11—C12—C13	179.86 (18)
Ni1—N2—C4—C1^i^	42.93 (14)	C14—N4—C13—C12	0.4 (3)
C3—N3—C5—C6	−160.49 (18)	C11—C12—C13—N4	−0.7 (3)
C2—N3—C5—C6	60.9 (2)	C13—N4—C14—C10	0.4 (3)
N3—C5—C6—C7	178.71 (18)	C11—C10—C14—N4	−0.8 (3)

Symmetry code: (i) −*x*+1, −*y*+1, −*z*+1.

### Hydrogen-bond geometry (Å, º)

**Table:**

*D*—H···*A*	*D*—H	H···*A*	*D*···*A*	*D*—H···*A*
N1—H1···O2	1.00	1.98	2.892 (2)	150
N2—H2···N4^ii^	1.00	2.23	3.143 (2)	151

Symmetry code: (ii) −*x*+1, −*y*+1, −*z*.
